# Establishment and Maintenance of Heat-Stress Memory in Plants

**DOI:** 10.3390/ijms25168976

**Published:** 2024-08-18

**Authors:** Shuzhi Zheng, Weishuang Zhao, Zimeng Liu, Ziyue Geng, Qiang Li, Binhui Liu, Bing Li, Jiaoteng Bai

**Affiliations:** 1 Ministry of Education Key Laboratory of Molecular and Cellular Biology, Hebei Collaboration Innovation Center for Cell Signaling and Environmental Adaptation, Hebei Basic Research Center of Cell Biology, Hebei Key Laboratory of Molecular and Cellular Biology, College of Life Sciences, Hebei Normal University, Shijiazhuang 050024, China; 2 Dryland Farming Institute of Hebei Academy of Agricultural and Forestry Science, Key Laboratory of Crop Drought Tolerance Research of Hebei Province, Hengshui 053000, China

**Keywords:** plant, heat-stress memory, chromatin accessibility, histone modification, signal transduction

## Abstract

Among the rich repertoire of strategies that allow plants to adapt to high-temperature stress is heat-stress memory. The mechanisms underlying the establishment and maintenance of heat-stress memory are poorly understood, although the chromatin opening state appears to be an important structural basis for maintaining heat-stress memory. The chromatin opening state is influenced by epigenetic modifications, making DNA and histone modifications important entry points for understanding heat-shock memory. Current research suggests that traditional heat-stress signaling pathway components might be involved in chromatin opening, thereby promoting the establishment of heat-stress memory in plants. In this review, we discuss the relationship between chromatin structure-based maintenance and the establishment of heat-stress memory. We also discuss the association between traditional heat-stress signals and epigenetic modifications. Finally, we discuss potential research ideas for exploring plant adaptation to high-temperature stress in the future.

## 1. Introduction

The stress memory of plants plays a crucial role in their adaptation to the environment [1]. Among these, heat-stress memory is an important strategy for plants to adapt to variable temperature conditions. In recent years, research on the molecular mechanisms underlying plant heat-stress memory has made some progress. Therefore, this section will introduce the biological functions of heat-stress memory and the advancements in the study of its molecular mechanisms.

### 1.1. Heat-Stress Memory: An Important Strategy for Plant Adaptation to the Environment

High-temperature stress is one of the most important abiotic stress factors affecting plant growth and crop yields worldwide [2,3,4]. Plants have evolved a series of defense mechanisms to adapt to such stress [5,6,7,8]. Among these adaptive strategies are basic thermotolerance, which refers to the inherent capacity of plants to withstand moderate heat; acquired thermotolerance, which is induced through exposure to sub-lethal heat-stress and confers enhanced resistance to subsequent stress events; heat-stress memory, an emerging concept that describes the plant’s ability to maintain a state of heightened tolerance following a stressful encounter [9,10]. Heat-stress memory is a dynamic and intricate process that is central to the maintenance of heat tolerance in plants. This phenomenon enables plants to not only survive but also to continue to grow and develop under conditions of external high-temperature stress, including when exposed to repeated episodes of high temperatures. Exploring the mechanism of heat-shock memory sheds light on the specific mechanism of plant adaptation to high-temperature stress and provides a theoretical basis for improving the heat tolerance of crops.

**Figure 1:**
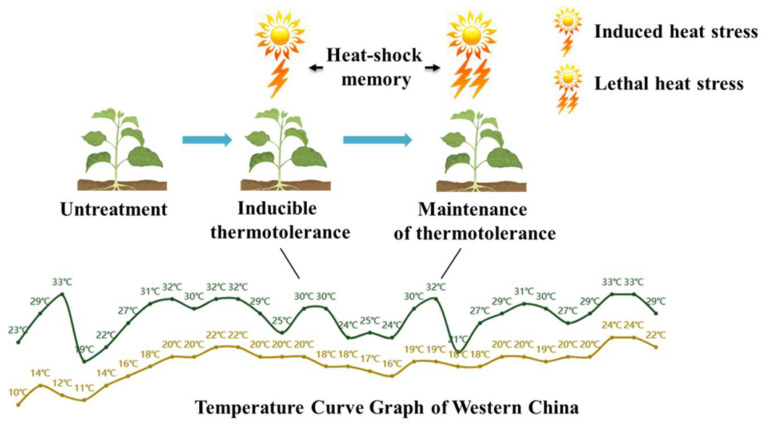
Diagram of heat-shock memory in plants. Ambient temperatures exhibit variability, with high temperatures typically manifesting indirectly. Moderately elevated temperatures can induce thermotolerance in plants, thereby enhancing their heat resistance. This capacity allows for survival upon subsequent exposure to higher temperatures. The plant’s ability to retain this heat resistance is referred to as thermotolerance memory. The green curve indicates day temperature, yellow curve indicates night temperature.

Heat acclimation involves relatively mild heat-stress in which plants are pretreated with a temperature of approximately 37 °C (for *Arabidopsis thaliana*). This treatment induces the expression of genes related to heat-shock protein synthesis, redox regulation, plant hormone regulation, calcium signaling, autophagy, and other metabolic pathways, which, in turn, increases plant tolerance to high temperatures [11,12,13]. These internal changes are “remembered” by the plant. Heat-shock memory reflects the ability of a plant to maintain this high-temperature tolerance (Figure 1). However, its mechanism is complex, and understanding the establishment and maintenance of heat shock memory can help us better understand the mechanisms by which plants adapt to high-temperature environments and provide insights for crop improvement. In conclusion, the adaptation of plants to high-temperature variations is a systemic event that is contingent upon the developmental state of the plant. This determines whether the plant will decelerate growth to withstand the stress period or expedite the transition to the reproductive phase. Consequently, an in-depth investigation of the molecular mechanisms underlying plant stress memory is essential for our understanding of plant growth and development in high-temperature environments.

### 1.2. Mechanism of High-Temperature Stress Memory in Plants

Prior exposure to stress may alter a plant’s subsequent stress response, producing a faster or stronger reaction that may increase plant stress tolerance. This response to consecutive stress indicates that plants exercise a form of stress memory. The heat-shock memory response reflects the ability to maintain tolerance to high temperatures. To date, research on the regulation of heat-shock memory has mainly focused on three aspects: the transcriptional regulation of genes, the stability of heat-shock proteins, and epigenetic modification [5,14].

The transcriptional regulation of genes primarily involves activation by transcription factors [15], differential gene expression [14,16], and differences in alternative splicing [17]. Its mechanism is complex and is influenced by various factors such as light [12,18], oxidative stress levels [19], and autophagy processes [13,20].

Multiple heat-shock factors and heat-shock proteins involved in heat-shock memory have been identified, including HSP18.2, HSP21, HSP22.0, HSP17.6c, and HSP101 among the heat-shock proteins and HSFA1, HSFA2, HSFA3, and HSA32 among the heat-shock factors (Table 1). For example, following heat-shock treatment, *HSP101* transcript levels rapidly decreased (within 24 h), but HSP101 protein levels remained high until 48 h of treatment had elapsed [21,22,23], highlighting the importance of heat-shock protein stability in regulating heat-shock memory. Heat-shock factors also affect histone modifications at the loci of heat-shock protein genes. For example, the loss of function of HSFA2 led to decreased histone H3 lysine 4 trimethylation (H3K4me3) modification at the *HSP22* gene locus [24].

Environmental stress induces epigenetic changes, such as DNA methylation and histone modification, that alter the chromatin structure and gene expression [25,26,27]. Epigenetic modifications play important roles in regulating chromatin accessibility and plant stress resistance. Extrinsic biotic and abiotic stress do not immediately alter the DNA sequences of plants. Therefore, epigenetic modifications are one of the mechanisms by which plants respond quickly to external stress [28,29,30]. Epigenetic regulation includes variations in nucleosome occupancy [31], histone modifications [32], chromatin remodeling [33], DNA methylation [34,35], and regulation by non-coding RNAs [36,37,38]. Epigenetic changes can produce stable memories and stress-induced gene expression [39,40,41]. Therefore, epigenetic modifications are another important mechanism of heat-shock memory [14,42]. During the maintenance of acquired thermotolerance, epigenetic modifications at heat-shock memory-related gene loci undergo changes, leading to the sustained expression of heat-shock memory-related genes. Stable epigenetic changes can even be transmitted across generations, maintaining stress memory for a long time, which allows plants to adapt to environmental stress [41,43,44]. Epigenetic modifications involved in heat-shock stress primarily include modifications of DNA and histones, such as DNA methylation, histone acetylation/deacetylation, and histone methylation/demethylation. Additionally, epigenetic modifications can induce structural changes in plant chromatin, thereby altering chromatin accessibility and providing a structural basis for the effects of these modifications on gene expression [45]. Therefore, the use of epigenetic modifications to activate enhancers can help guide gene function to improve crop traits. These epigenetic modifications shape plant stress plasticity, and their cumulative effects enhance plant adaptation to high-temperature stress [40].

Mitochondrial adaptation is one of the crucial mechanisms by which plants adjust to stressful environments. Mitochondrial adaptation refers to the response and adjustment of mitochondria within plant cells to various environmental conditions or changes in physiological states [46]. Although there is currently no direct evidence demonstrating a regulatory relationship between mitochondrial adaptation and the memory of heat-stress, the existing reported experimental results suggest that the relationship between the two remains a research direction worthy of attention [47].

Current methodologies for the study of heat-stress memory encompass techniques such as targeted gene mutation [48,49], large-scale phenotypic screening, construction, analysis of multiple mutant strains [50], and high-throughput sequencing [51]. However, each of these research technologies has its limitations. Future studies that achieve breakthroughs in genetic technology could potentially provide novel perspectives for the investigation of the mechanisms underlying heat shock memory. In conclusion, the establishment and maintenance of plant heat-stress memory is a systemic event. Current research primarily focuses on specific levels of study, with a deficiency in correlative analysis across different levels. Future research endeavors that explore the relationships between transcription factors, transcription, gene modification, and genetics may provide novel avenues for future research (Table 1).

**Table 1:** Summary of regulators of heat-stress memory in plants.

Gene	Annotation	Description	References
Heat-shock proteins and heat-shock factors			
*HSA32*	Non-canonical heat-shock protein		[52]
*HSFA2*	Heat-shock transcription factor	Reduces H3K4me3 at *HSP22*	[14,50]
*HSP22/HSP17.6C*	Small heat-shock proteins	Decrease or increase in chaperone function	[53]
*HSFA1*			[54]
*HSP21/HSP22.0/HSP18.2*	Small heat-shock proteins	Decrease or increase in chaperone function	[55]
*HSP101*	Heat-shock protein	Decrease in chaperone function	[21]
*HSFA2/HSFA3*	Heat-shock factors	Accessible chromatin environment, and heat-stress-induced enrichment of H3K4me3	[25,26]
*HSP101*	Heat-shock protein	HSP101 positively regulates *HSA32* levels	[56]
Chromatin remodeler and nucleosome occupancy			
*CHR11/CHR17*	SWI2/SNF2 chromatin remodelers	Increases nucleosome occupancy at *HSA32*	[31]
*BRM*	SWI2/SNF2 chromatin remodeler	Increases nucleosome occupancy at *HSA32*	[31]
*FGT1*	Ortholog of Strawberry notch	Interacts with chromatin remodelers of the SWI/SNF and ISWI families	[31]
Histone acetyltransferases/deacetylase in thermotolerance			
*FGT2*	Type 2C protein phosphatase	Changes in lipid homeostasis	[57]
*PLDA2*	Phospholipase D α2	Changes in lipid homeostasis	[57]
*ROF1*	Peptidyl prolyl cis/trans isomerase, A member of the FKBP family	HsfA2-regulated expression of small *HSP* genes is greatly reduced in *rof1*	[58]
*HDA9-PWR-ABI4*	Proteins complex	Promotes drought tolerance through deacetylation	[30,33]
*FtsH6*	FtsH metalloprotease	Promotes *HSP21* accumulation	[55]
Histone methylation/demethylation in thermotolerance			
*TFB5*, *CHMP1B*, *RSZ22*, *CHLM*, *RZ1A*, *HDT2*	*Pinus* proteins	Long-term heat-stress splicing memory	[59]
*NBR1*	A receptor for selective autophagy during recovery from HS	NBR1 interacts with HSP90.1 and ROF1 and mediates their degradation by autophagy,	[60]
*CSN5A*	CSN5A subunit of the COP9 signalosome	resetting transcriptional memory genes (*APX2* and *HSP22*) and H3K4me3 following recurrent heat-stress	[61]
*ATX1*	H3K4 methyltransferase	Regulates H3K4me3 levels at the promoters of HS recovery genes	[62]
*JMJs*	H3K27me3 demethylases	High H3K27me3 at *HSP22*/*HSP17.6C*	[63]
*SDG25*, *ATX1*	Histone H3K4 methyltransferases	Decrease histone H3K4me3 levels and increase DNA cytosine methylation	[64]
*CAS*	Calcium sensing receptor (CAS) protein	*cas* mutants display enhanced biomass and reduced degradation of the small heat-shock protein HSP 17.6	[65]
Non-coding RNA			
*miR156/SPL*	MicroRNA/Transcription factors	Decrease or increase *SPL* mRNA levels	[66]
*AGO1*	RNA slicer	High *miR156* levels	[66]
*DCL1*	Dicer	High *miR156* levels	[66]
Autophagy in thermopriming			
*BRU1*	Tetratricopeptide-repeat (TPR) and leucine-rich repeat (LRR) protein interactor	The *bru1* mutant shows strong induction of *HSA32* during recovery	[67]
*HLP1*	Ortholog of human *Hikeshi*	Accumulation of 3K4me3 at thermomemory-associated loci	[68]
*TOR*	Target of rapamycin	TOR promotes long-term accumulation of H3K4me3 on thermomemory-associated gene promoters	[62]
*FORGETTER2*	Type 2C protein phosphatase (PP2C) of the D-clade	FGT2 interacts with phospholipase D α2 (PLDα2)	[57]

## 2. Establishment of Heat-Stress Memory: Calcium May Be a Key Signal

### 2.1. The Relationship between Heat Shock Signal Transduction and Heat Shock Memory

Plants often experience slower growth and development when they are kept in a stressful state. Therefore, understanding what signals cause plants to have memories is crucial for plants’ survival and reproduction. However, so far, the understanding of the formation of heat shock memory is still insufficient. The traditional calcium-dependent heat-shock signaling transduction pathway has been well studied [69,70]. This pathway allows plants to respond quickly to heat-stress and establishes heat-shock memory via the production of HSPs. However, few studies to date have linked the maintenance of heat-shock memory through heat-shock signaling transduction to chromatin structures in plants [14,25,71,72]. Future studies should aim to elucidate the molecular dialogue between calcium signaling pathways and chromatin-modifying enzymes. This could involve investigating how calcium influx affects the recruitment of histone-modifying enzymes or chromatin remodeling complexes to stress-responsive genes. Additionally, the temperature sensor is at the most upstream position of the cell to sense temperature signals, and common plant temperature sensors include PHYTOCHROME B (phyB) [73], EARLY FLOWERING 3 (ELF3) [74,75], PIF7, and Phot [76]. Exploring the regulatory relationship between temperature sensors and heat shock memory can promote the understanding of the establishment and maintenance mechanisms of “memory”.

### 2.2. The Relationship between Ca^2+^ and Heat-Stress Memory

Ca^2+^ signaling plays a critical role in sensing sudden changes in temperature and activates signaling cascades, leading to the production of heat-shock proteins that increase plant thermotolerance [70]. Currently, we are interested in the question, “Does the calcium ion signal establish heat shock memory by regulating chromatin accessibility to provide a structural basis for gene expression?” Although there is still limited evidence in plants, calcium (an early signal of heat-stress) may activate downstream chromatin opening states, enabling transcription factors and HSPs to function and further regulate chromatin accessibility [77,78,79]. Studies in animals have provided some experimental evidence for a relationship between the traditional heat-shock signaling pathway and chromatin accessibility. For example, in myocardial cells, calcium mediates histone modifications to regulate alternative splicing [80]. In GH3 cells, calcium signaling activates the expression of the prolactin gene, leading to chromatin opening and the expression of related genes. When the level of the calcium signal is low, the expression level of prolactin is also low, and the chromatin state is closed, blocking gene expression [81]. In tumor cells, the phosphorylation of histone H3 has long been known to be regulated by calcium [82].

However, the relationship between calcium and high-temperature stress in plants has not been deeply studied. However, in other types of abiotic stress processes, it seems that calcium signaling can regulate histone modification to affect plant stress resistance. In response to extracellular calcium, *Arabidopsis* PROTEIN ARGININE METHYLTRANSFERASE 5 (PRMT5) binds to the promoter of the calcium signaling gene calcium-sensing receptor (*CAS*), resulting in a decrease in histone H4 Arg 3 with symmetric dimethylation (H4R3sme2) modification, which, in turn, suppresses *CAS* expression and affects stomatal closure and the plant’s response to drought [77]. Moreover, the regulated G-protein-coupled receptor (GPCR) increases in calcium trigger nuclear-actin-dependent changes in the chromatin organization by promoting nuclear F-actin assembly for rapid responses via chromatin dynamics in plants [78].

Heat-induced increases in Ca^2+^ levels in chloroplasts function as a positive signal for the resetting of heat-stress memory in plants [65]. Moreover, [Ca^2+^]cyt-activation of S-type anion channels is primed by the pre-exposure of guard cells to abscisic acid, a process called Ca^2+^ sensitivity priming [83]. Cyclic-nucleotide-gated Ca^2+^ channels in the plasma membrane respond to increases in ambient temperature by triggering an optimal heat-stress response, leading to the onset of acquired thermotolerance [84]. Ca^2+^ enters the cell through calcium channels and primarily exerts its biological functions by binding to calmodulin (CaM). Calmodulin is a calcium-sensing receptor that is widely present in eukaryotic cells, modulating a range of physiological activities in plants by binding to various target proteins [85]. However, research on the relationship between heat shock memory and calmodulin is still very limited, and there is no direct evidence to prove a regulatory relationship between the two. Currently, only a few studies hint at a possible correlation between them. For example, the expression of the Calmodulin-like 41 (CML41) gene is up-regulated by a high temperature with reduced DNA methylation levels [86]. Another study has reported that SDG8 has been implicated in the epigenetic memory-forming process, inducing the expression of TOUCH3 (TCH3), which encodes a calmodulin-like protein (CML12) [87]. Additionally, CALMODULIN-BINDING PROTEIN KINASE 3 (CBK3) has been reported as an HsfA1-interacting protein kinase that regulates the DNA-binding capacity of HsfA1, a key switch in heat-stress memory [88]. In summary, although there is currently no sufficient direct evidence to prove that the calcium signal induced by high temperature can regulate histone modification to establish heat shock memory, there are still some experimental clues suggesting a possible relationship between the two. While many studies have been made to understand the initial steps of the heat shock response, the mechanisms that underpin the long-term maintenance of heat shock memory and its interplay with chromatin structure remain enigmatic. Unraveling these mechanisms is critical for us to understand the relationship between the rapid response to high-temperature stress mediated by calcium ions and the long-term maintenance of thermotolerance.

### 2.3. Ca^2+^ and Histone Modifications

Ca^2+^ is an important intracellular second messenger that regulates many biological processes during heat-stress. Although many studies have focused on how calcium regulates heat signal transduction, a few have also explored how calcium can lead to histone modifications. In animals, on the one hand, calcium can modify histones through phosphorylation; for example, Ca^2+^ ions promote the phosphorylation of histone H3 in HeLa cells [82]. On the other hand, calcium ions can also regulate alternative splicing; for example, calcium-mediated epigenetic changes regulate gene expression at the level of alternative splicing in cardiomyocytes [80]. In plants, in response to increases in calcium, fewer CAU1 (also named PRMT5) protein molecules bind to the *CAS* promoter, leading to a decrease in symmetric dimethylation of histone H3 arginine 3 (H4R3sme2) and the derepression of *CAS* expression to mediate drought tolerance [77]. In summary, calcium ions may regulate histone modification under high-temperature stress, but current evidence is limited, and further research is needed to confirm this hypothesis.

### 2.4. Transcription Factors, Heat Shock Factors and Heat-Stress Memory

Transcription factors and heat shock factors are important components in the traditional signal transduction process. When histones in nucleosomes disassemble, transcription factors competitively bind to DNA sites, increasing openness in the region’s chromatin and providing conditions for the addition of other transcription factors or co-factors. Transcription factors initially bind to linker DNA between nucleosomes, further disrupting adjacent nucleosomes through competitive binding or with the help of remodeling complexes, thereby establishing chromatin accessibility in the region [89].

The expression of *HSFA2* is directly regulated by HSFA1 and is also involved in the establishment of heat shock memory. HSFA2 binds to heat-shock memory genes after heat-stress [90], while these memory genes exhibit changes in H3K4me2/3 modification for at least several days [14]. Although both HSFA2 binding and H3K4me2/3 sustained accumulation have been observed on heat-shock memory genes, the regulatory relationship between the two remains unclear.

HSFA3 is specifically required for physiological heat-shock memory and for maintaining high memory-specific gene expression during the days following HS exposure. HSFA2 and HSFA3 efficiently promote transcriptional memory by positively influencing histone H3K4 hyper-methylation [26]. HSFA2 directly activates H3K27me3 demethylase REF6, which, in turn, derepresses *HSFA2*. REF6 and HSFA2 form a heritable feedback loop that orchestrates transgenerational thermomemory in *Arabidopsis* [91]. In *Arabidopsis*, heat-shock memory is associated with the accumulation of H3K4me2/3 at memory-related loci. Transcriptional memory and the sustained accumulation of H3K4 methylation depend on HSFA2. HSFA2 is transiently associated with memory-related loci during the early stages following heat-stress [14]. Finally, the transcription factor gene *JUB1* shows thermomemory-related expression and a *jub1* mutant shows reduced heat-stress memory [92]. Taken together, the expression of heat shock memory genes depends on both the transcription factors and the histone modifications at the genomic loci. This synergistic regulation at the chromatin and protein levels allows for more precise control.

## 3. Maintenance of Heat-Stress Memory: Chromatin Accessibility Is a Key Structural Basis

### 3.1. Chromatin Accessibility: A Structural Foundation of Stress Memory

In recent years, growing evidence has demonstrated important functions of chromatin accessibility and epigenetic histone modifications in regulating gene expression, representing important mechanisms for plant adaptation to heat-stress [25]. When plants are exposed to heat-stress, the three-dimensional structure of chromatin changes; chromatin opening is the structural basis for the regulation of gene transcription and for maintaining heat tolerance [54,93]. Heat-stress affects 3D chromatin architecture, including the A/B compartment transition, the topologically associated domain (TAD) size, and long-range interactions. Therefore, chromatin accessibility provides a new layer in our understanding of the transcriptional regulation of plant responses to heat-stress [93]. These changes enable plants to express genes more rapidly upon re-exposure to high-temperature stress.

In plants, genomic DNA is gradually compressed to form chromatin by binding to histones and histone chaperones [94,95]. However, the precise regulation of gene expression can occur quickly in plant cells [96,97]. This indicates that chromatin is highly dynamic; in addition, its assembly follows specific rules. Moreover, an efficient chromatin regulatory pattern in plant cells facilitates the precise regulation of gene expression [98,99].

Most regions of eukaryotic chromatin that do not exhibit transcriptional activity are highly compressed, but some open chromatin remains; the degree to which chromatin is accessible to regulatory proteins is called chromatin accessibility [89,100,101]. The establishment and maintenance of chromatin accessibility are influenced by many factors, such as DNA methylation [102], nucleosome positioning [103], transcription factor binding [89], and chromatin remodeling factors [104,105]. Chromatin accessibility in cells undergoes dynamic changes during growth [106], development [107,108], and responses to external stimuli [93,109]. All of these processes, which rely on the chromatin structure or directly affect chromatin accessibility to regulate gene expression, are crucial for plant adaptation to environmental factors, and the precise regulation of gene expression modulates cell proliferation, differentiation, and cell death, thereby maintaining plant growth, development, and adaptation [64,110,111,112,113,114]. In high-temperature environments, plants may exhibit distinct mechanisms for maintaining chromatin accessibility at different developmental stages and growth conditions and elucidating these mechanisms represents an important aspect of future research.

### 3.2. Nucleosome Positioning and Heat-Stress Memory

Nucleosome positioning is also altered in response to heat-stress [115]. The organization of nucleosomes in the genome is not static but is instead dynamically changing. These dynamic changes are manifested by the dynamic sliding of nucleosomes on DNA, which spontaneously undergo partial or complete disassembly [116]. Nucleosomes have different rates of disassembly in different chromatin regions, with significantly higher rates in active promoters and enhancers than in inactive heterochromatin. The positioning and disassembly of nucleosomes are related to many regulatory factors, including DNA sequence specificity and the participation of histone variants, chromatin remodeling complexes, molecular chaperones, and transcription factors. AtASF1A/B are involved in the heat-stress response in plants and participate in the transcriptional activation of certain HSF and HSP genes via nucleosome removal and the stimulation of histone H3 lysine 56 acetylation (H3K56ac) [72]. FGT1 interacts with chromatin remodelers of the SWI/SNF and ISWI families, thereby modulating nucleosome occupancy and mediating heat-stress-induced chromatin memory [31]. Therefore, plants may remember past stress events by maintaining or “memorizing” specific nucleosome positioning patterns, which helps them to respond more rapidly to future stresses.

### 3.3. Enhanced Chromatin Accessibility through Histone Acetylation Modification

The histone acetylation is involved in the thermal stress response. Histone acetylation promotes gene expression by neutralizing the positive charges of lysine residues, thereby reducing the charge-dependent interaction between histones, nucleosome DNA, linker DNA, or adjacent histones. This reduces chromatin compaction, enhances the accessibility of chromatin DNA to the transcriptional machinery, and promotes the transcription of genes in the region. In the nematode *Caenorhabditis elegans*, the histone acetyltransferase CBP-1 and the chromatin remodeling SWI/SNF complex are epigenetic modulators of long-lasting defense responses to promote longevity following exposure to heat-stress during early life [117]. In plants, the HAT family protein GCN5 plays an essential role in promoting thermotolerance. GCN5 enhances acetylation, specifically H3K9ac and H4K14ac, in the promoter regions of *HSFA3* and *ULTRAVIOLET HYPERSENSITIVITY6* (*UVH6*), and the modification is associated with their enhanced expression and enhanced thermotolerance in *Arabidopsis* [71]. Moreover, there is an HDA9-mediated positive regulatory module in the heat shock signal transduction pathway due to cytoplasm-to-nucleus translocation in the plants [118]. This translocation and subsequent chromatin remodeling represent a dynamic and responsive mechanism by which plants can adjust their transcriptional programs in response to thermal cues.

### 3.4. Histone Methylation and Heat-Stress

Histone methylation also affects chromatin accessibility [119]. H3K9me3 and H3K27me3 are associated with the formation of heterochromatin and the silencing of the Polycomb inhibitory complex, respectively [120]. Methylation increases the affinity of specific protein modules for histone residues, further enhancing the stability of nucleosomes. However, histone methylation does not always reduce the accessibility of chromatin. For example, H3K4me3 is a marker of transcriptional activation that provides attachment sites for transcription factors, further recruiting RNA polymerase to enhance the chromatin opening. H3K4me3 is mainly enriched at the promoters/transcription start sites of the genes. However, even inactive promoters can demonstrate the enrichment of H3K4me3; these sites are typically potential or soon-to-be active promoter/transcription start sites, indicating that this modification enhances chromatin accessibility [121].

**Figure 2:**
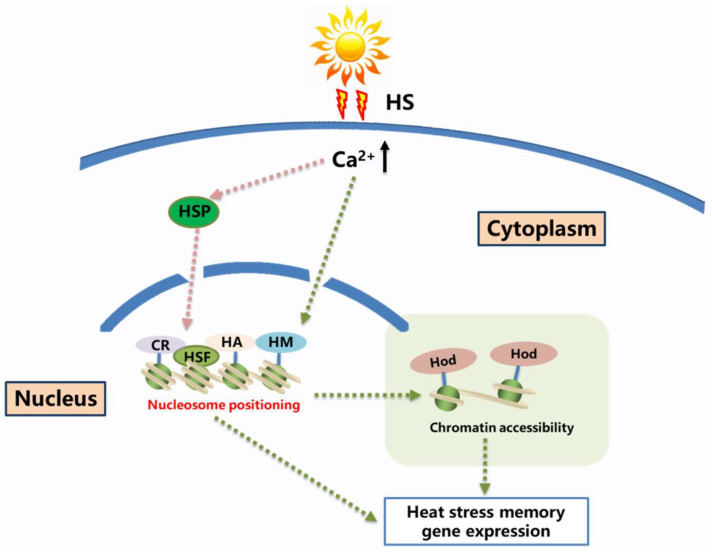
Signaling networks regulating plant heat shock memory. Upon heat shock, the intracellular calcium ion concentration in plants increases rapidly, serving as a crucial upstream signal for the expression of heat shock genes. The role of calcium ions in influencing the modification of heat shock proteins and chromatin is not yet well understood. Chromatin modifications include histone acetylation and methylation, which lead to chromatin opening and, consequently, the regulation of heat shock gene expression. HS: Heat shock; HA: Histone acetylation; HM: Histone methylation; NP: Nucleosome positioning; CR: Chromatin remodeler; HSF: Heat-shock transcription factor; HSP: heat-shock proteins; HOD: Histone modification.

Moreover, H3K4me2/3 plays important roles in plant responses to heat-stress [14], cold stress [122], and drought stress [123]. After heat-shock treatment, H3K4me2/3 modifications are enriched at heat-shock memory genes. Histone methylation modifications are regulated by the SET (Su(var), Enhancer of zeste (E(z)), and Trithorax) domain-containing proteins, which participate in chromatin condensation and separation, transcriptional regulation, DNA replication and repair, and other cellular processes, playing important roles in plant development and stress tolerance. The *ARABIDOPSIS TRITHORAX (ATX)* family is an important SET-domain-containing methyltransferase family with five members: ATX1, ATX2, ATX3, ATX4, and ATX5. ATX3, ATX4, and ATX5 share high homology and functional redundancy and specifically participate in H3K4me2/3 methylation modifications [124]. These results indicate that the functions of histone methylation are not only diverse but also exhibit a certain level of complexity in their regulation (Figure 2).

## 4. Challenges and Perspectives

The establishment of the chromatin state is closely related to stress memory, but research in this area is still insufficient [27]. The specific mechanism by which plant cells sense changes in environmental temperature, especially high temperatures, is not yet fully understood [125]. Whether the degree of chromatin opening is related to traditional heat-shock signaling pathways is worth exploring; although there are some experimental clues [91], in-depth research is still lacking. High-throughput sequencing technologies for detecting chromatin openings have been rapidly developing in recent years, providing a technological basis for exploring the biological functions of chromatin openings [126,127].

There has been some investigation of histone acetylation and methylation modifications in response to high temperatures and other types of stress. However, it remains to be determined whether other novel modification types (such as histone monoubiquitination, sumoylation, and nitrosylation) are involved in the establishment and maintenance of heat-shock memory [128,129,130]. In addition, different modification types may also cooperatively regulate chromatin openings, allowing for the more precise regulation of gene expression [131].

It will also be important to determine how long chromatin openings can be maintained during plant adaptation to environmental changes [64]. Based on limited evidence, it appears that plants may have two regulatory mechanisms for chromatin openings—long-term memory and short-term memory [91,132,133,134,135]—that play different roles in plant environmental adaptation [136,137,138]. Indeed, the mechanisms for the establishment and maintenance of long-term and short-term memory may differ; further research is needed to understand how these processes are triggered or transformed [139].

## 5. Conclusions

In this review, we summarized the current understanding of the mechanisms underlying heat-shock memory, the regulation of chromatin openings, and the establishing mechanisms of heat-stress memory. We discussed the interplay between traditional heat-shock signaling pathway components, including Ca^2+^ and transcription factors and chromatin openings, and reviewed the relationships between chromatin accessibility and calcium ions, heat-shock factors, and heat-shock proteins. Calcium ions (Ca^2+^) are crucial secondary messengers in plants, involved in sensing environmental changes and activating downstream responses. The relationship between calcium ion signal transduction and chromatin accessibility is currently not well understood. Calcium-dependent modulation could potentially provide a structural basis for the expression of heat-shock proteins and other stress-responsive genes, which are critical for the establishment of heat-stress memory. By studying the interplay between Ca2+ signaling and chromatin accessibility, researchers can gain a deeper understanding of the epigenetic and molecular mechanisms that underpin stress memory in plants. This knowledge could potentially be harnessed to develop strategies for improving crop resilience to environmental stressors, such as high temperatures, which are becoming increasingly prevalent due to climate change.
